# The Phosphorylation-Dependent Regulation of Mitochondrial Proteins in Stress Responses

**DOI:** 10.1155/2012/931215

**Published:** 2012-07-15

**Authors:** Yusuke Kanamaru, Shiori Sekine, Hidenori Ichijo, Kohsuke Takeda

**Affiliations:** ^1^Laboratory of Cell Signaling, Graduate School of Pharmaceutical Sciences, The University of Tokyo, 7-3-1 Hongo, Bunkyo-ku, Tokyo 113-0033, Japan; ^2^Division of Cell Regulation, Nagasaki University Graduate School of Biomedical Sciences, 1-14 Bunkyo-machi, Nagasaki 852-8521, Japan; ^3^Precursory Research for Embryonic Science and Technology (PRESTO), Japan Science and Technology Agency (JST), 4-1-8 Honcho Kawaguchi, Saitama 332-0012, Japan

## Abstract

To maintain cellular homeostasis, cells are equipped with precise systems that trigger the appropriate stress responses. Mitochondria not only provide cellular energy but also integrate stress response signaling pathways, including those regulating cell death. Several lines of evidence suggest that the mitochondrial proteins that function in this process, such as Bcl-2 family proteins in apoptosis and phosphoglycerate mutase family member 5 (PGAM5) in necroptosis, are regulated by several kinases. It has also been suggested that the phosphorylation-dependent regulation of mitochondrial fission machinery, dynamin-related protein 1 (Drp1), facilitates appropriate cellular stress responses. However, mitochondria themselves are also damaged by various stresses. To avoid the deleterious effects exerted by damaged mitochondria, cells remove these mitochondria in a selective autophagic degradation process called mitophagy. Interestingly, several kinases, such as PTEN-induced putative kinase 1 (PINK1) in mammals and stress-responsive mitogen-activated protein (MAP) kinases in yeast, have recently been shown to be involved in mitophagy. In this paper, we focus on the phosphorylation-dependent regulation of mitochondrial proteins and discuss the roles of this regulation in the mitochondrial and cellular stress responses.

## 1. Introduction

 Mitochondria play a fundamental role in cells, serving as the “powerhouses” that produce ATP through the process of oxidative phosphorylation. In addition to supplying cellular energy, mitochondria are involved in the response to several cellular stresses, such as cell death signaling and antiviral immunity [1–3]. However, mitochondria themselves are also exposed to various stresses. For example, leakage of the high-energy electrons in the respiratory chain leads to the formation of reactive oxygen species (ROS), which can damage mitochondrial DNA (mtDNA). Mutations in mtDNA result in enzymatic abnormalities in the mitochondrial respiratory chain and further oxidative stress. This vicious cycle has been considered to be involved in a wide range of human diseases, such as metabolic, aging, and neurodegenerative diseases [4]. Thus, the quality of mitochondria must be continuously monitored and maintained by various strategies.

 Mitochondria are dynamic organelles that constantly fuse and divide [5]. These dynamic properties are important for the maintenance of mitochondrial functions, which ultimately contribute to cell survival. A certain degree of mitochondrial damage, including mtDNA mutations, can be rescued by mitochondrial fusion, which allows mitochondrial content mixing within a cell (see Section 2). However, severe mitochondrial damage impairs their fusion and result in mitochondrial fragmentation. The resulting fragmented mitochondria are then selectively removed by an autophagic process, termed mitophagy (see Section 3). In other cases, when cells are critically damaged, the mitochondria act as a hub of cell death pathway signaling (see Sections 4 and 5). Several apoptosis-promoting factors are released from mitochondria, and many apoptosis-regulating proteins exert their roles on mitochondria; thus, it has been well documented that mitochondria have crucial roles in apoptosis [2]. In addition, recent studies have revealed that mitochondria also contribute to necroptosis, another type of cell death [6].

 Recently, the development of new mass spectrometry-based technologies has led to the discovery of many novel phosphorylation sites on a variety of mitochondrial proteins [7]. Interestingly, it has been suggested that the phosphorylation-dependent regulation of mitochondrial proteins appear to have important roles in mitochondrial and cellular stress responses. In this paper, we will summarize recent research related to mitochondria, especially focusing on the phosphorylation-dependent regulation of various mitochondrial proteins.

## 2. Mitochondria Are Dynamic Organelles

**Figure 1:**
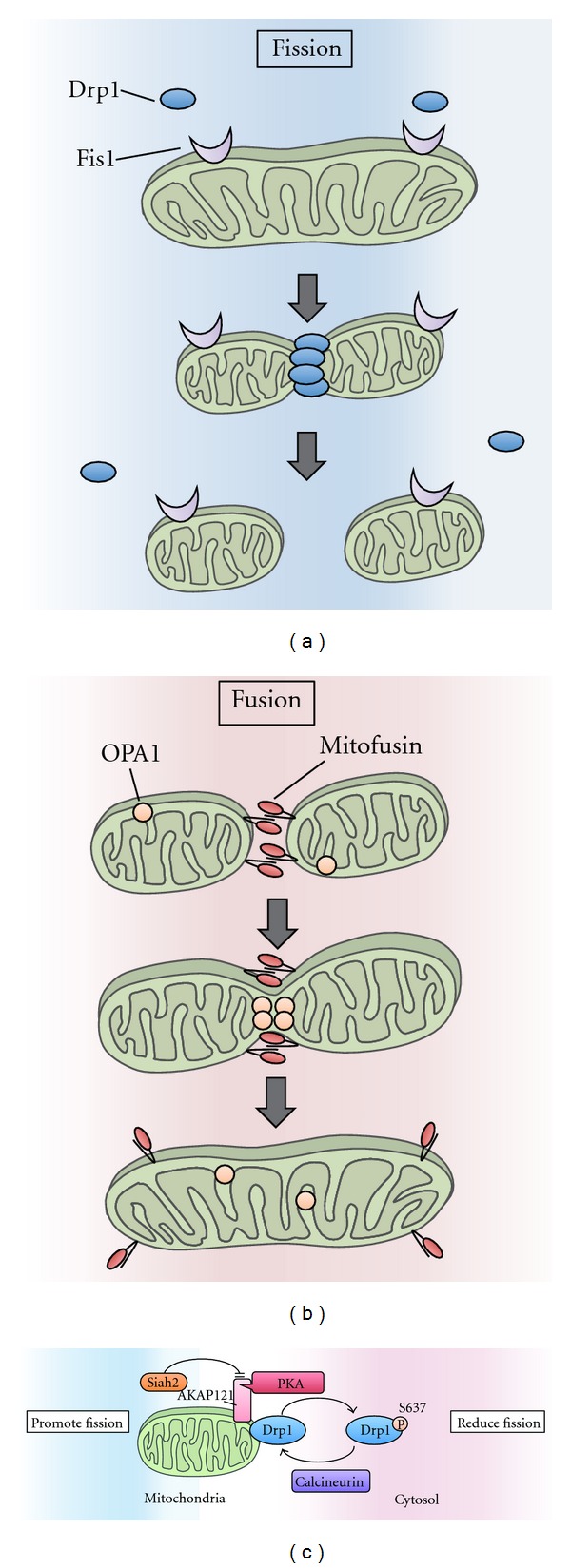
Mitochondrial fission and fusion. (a) Mitochondrial fission is driven by Drp1. Drp1 is recruited to the mitochondrial outer membrane at the fission sites and promotes mitochondrial fission. Fis1 resides on the mitochondrial outer membrane and is considered to function as a receptor for Drp1. (b) Fusion is driven by OPA1 and mitofusins. Interactions between these proteins tether two adjacent mitochondria. Mitofusins mediate mitochondrial outer membrane fusion, while Opa1 mediates mitochondrial inner membrane fusion. (c) The mitochondrial fission-promoting activity of Drp1 is controlled by the phosphorylation of Drp1 at Ser637. PKA phosphorylates Drp1 and inhibits the translocation of Drp1 to mitochondrial fission sites. Conversely, the calcineurin-mediated dephosphorylation of Drp1 results in the recruitment of Drp1 to the mitochondria and promotes mitochondrial fission. AKAP121 is a mitochondria-localized adaptor protein that regulates mitochondrial dynamics by facilitating Drp1 phosphorylation via PKA anchoring on mitochondria. Siah2 promotes the degradation of AKAP121, which decreases Drp1 phosphorylation, thus resulting in mitochondrial fission.

 Mitochondria are highly dynamic organelles that constantly change shape and form flexible reticular networks [5]. Mitochondrial morphology is controlled by a balance of fusion and fission. These events are known to be regulated by the members of the dynamin superfamily of large GTPases, specifically dynamin-related protein 1 (Drp1), the mitofusins MFN1 and MFN2, and optic atrophy type I (OPA1) (Figures 1(a) and 1(b)). Drp1 is a cytosolic protein that is involved in mitochondrial fission, whereas OPA1 and mitofusins reside in mitochondria and mediate their fusion. The recruitment of Drp1 from the cytosol to mitochondrial fission sites involves Fis1, a mitochondrial integral outer membrane protein that is essential for fission. Why do mitochondria continually fuse and divide? Recent studies have shown that these events are required to maintain functional mitochondrial populations in the cell. For example, fusion is thought to allow the exchange of contents between intact and dysfunctional mitochondria. This “content mixing” allows the replacement of damaged material, such as mtDNA that harbors mutations, contributing to the suppression of further damage and mitochondrial homogeneity [8, 9]. In contrast, fission is thought to allow the segregation of severely damaged mitochondria from healthy mitochondrial networks [10]. These segregated damaged mitochondria are delivered to autophagosomes and ultimately degraded (see Section 3 for details). Consistent with these implications of physiological importance of mitochondrial dynamics, the knockout of any of these genes in mice causes embryonic lethality [11–13]. In addition, some mitochondrial genes are known to cause some types of neurodegenerative diseases. Mutations in *Mfn2* cause the Charcot-Marie-Tooth type 2A (CMT2A) [14], one of the most common hereditary neuropathies, and mutations in *OPA1* are the predominant cause of autosomal dominant optic atrophy (DOA), a heritable form of optic neuropathy [15, 16]. These lines of genetic evidence indicate that the dynamic properties of mitochondria may have important roles, particularly in neurons [5].

### 2.1. Drp1 Phosphorylation

In contrast to mitochondria-localized OPA1 and mitofusins, the majority of Drp1 protein is cytoplasmic [5]. Thus, the localization of Drp1 to mitochondria has emerged as a key regulatory step for mitochondrial fission. Recent studies have suggested that the phosphorylation of Drp1 at Ser637 is an important regulatory modification. This phosphorylation has been reported to be regulated by the opposing actions of PKA, cAMP-dependent protein kinase [17], and calcineurin, a Ca2^+^-and calmodulin-dependent protein phosphatase [18]. This modification appears to inhibit mitochondrial fission by inhibiting Drp1 recruitment to mitochondria and/or the reduction of its GTPase activity (Figure 1(c)) [17, 18].

### 2.2. The Control of Mitochondrial Dynamics during Stress Response through the Phosphorylation of Drp1

Several lines of evidence revealed that regulated changes in mitochondrial morphology through the phosphorylation of Drp1 at Ser637 determine cell fate under various stresses, including starvation and hypoxia [19, 20].

 During starvation, mitochondria were reported to elongate through the PKA-mediated phosphorylation of Drp1 on Ser637 [19]. Because the genetic or pharmacologic blockade of mitochondrial elongation promotes starvation-induced cell death, mitochondrial elongation is considered to protect the cell against starvation. Although starvation is one of the most common inducers of a self-degradation system called macroautophagy, elongated mitochondria seem to be spared from autophagy. Retained mitochondria help to maintain the cellular ATP level, leading to sustained cell viability [19]. Mitochondrial hyperfusion has also been reported to protect against apoptosis under other stress conditions, such as UV irradiation and actinomycin D treatment, through a different set of molecular mechanisms [21]. These findings indicate that mitochondrial elongation is an important and effective stress response to ensure cell survival.

 In contrast, hypoxia induces mitochondrial fission by suppressing the phosphorylation of Drp1 on Ser637 [20]. The E3 ubiquitin ligase Siah2 and the mitochondrial scaffold protein AKAP121 are the key regulators of hypoxia-induced mitochondrial fission (Figure 1(c)). The A-kinase anchor protein (AKAP) family is composed of scaffold proteins that tether PKA and other signaling molecules to distinct subcellular organelles. AKAP121 is associated with mitochondria [22] and facilitates the PKA-mediated phosphorylation of Drp1 at the mitochondria [20]. Siah2 was originally identified as a hypoxia-responsive E3 ligase that ubiquitinates PHD enzymes [23]. PHD enzymes mediate the prolyl hydroxylation of hypoxia-inducible factor 1*α* (HIF1*α*), a central regulator of the cellular response to hypoxia. Because the prolyl hydroxylation of HIF1*α* is a prerequisite for its recognition by the von Hippel-Lindau protein (pVHL) and subsequent proteasomal degradation, the Siah2-mediated degradation of PHD relieves HIF1*α* from degradation and thus stabilizes HIF1*α* [23]. Interestingly, Siah2 also ubiquitinates AKAP121 and promotes its degradation under hypoxic conditions [20]. The reduced availability of AKAP121 suppresses the PKA-mediated phosphorylation of Drp1 and facilitates the Drp1-Fis1 interaction, resulting in mitochondrial fission. In cells lacking Siah2, hypoxia-induced fission is suppressed, and this is correlated with high AKAP121 levels and Drp1 phosphorylation in the mitochondrial fraction, ultimately leading to the suppression of hypoxia-induced cell death.

## 3. The Phosphorylation-Dependent Regulation of Mitophagy, a Mitochondrial Quality Control System

 As mentioned above, mitochondrial fusion is one mitochondrial quality control system. However, when mitochondria are severely damaged, other stress responses are induced [24].

**Figure 2:**
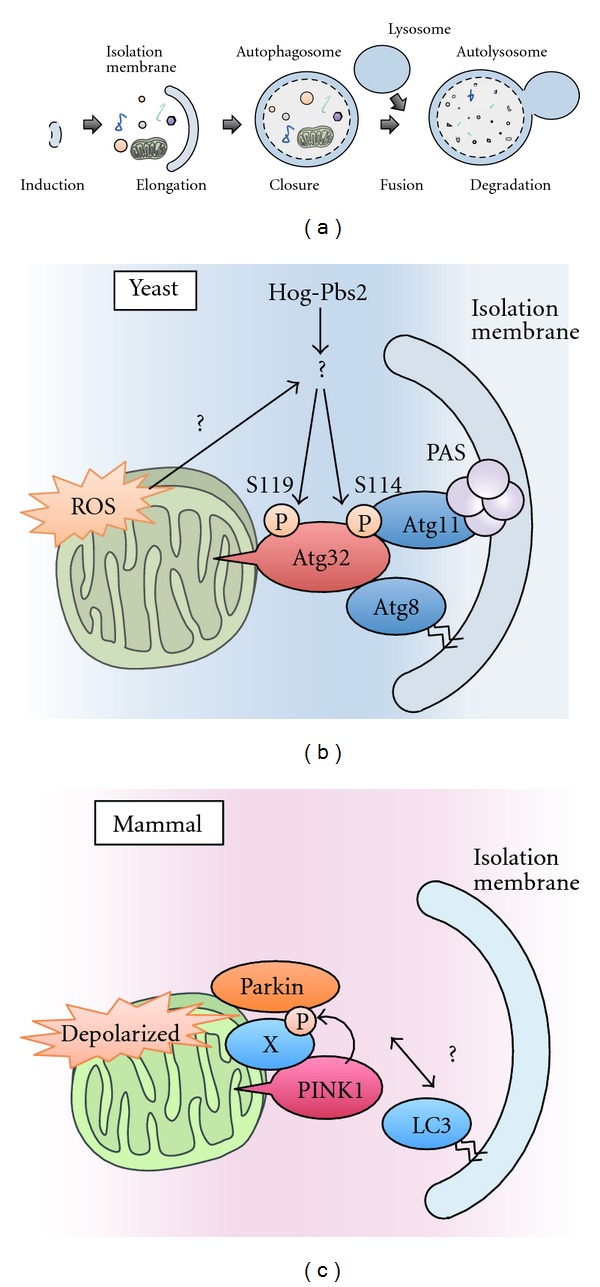
Mitophagy in yeast and mammals. (a) In autophagy, a double-membrane compartment, termed the isolation membrane, engulfs a portion of cytoplasm, including macromolecules and organelles, and forms autophagosomes. The late autophagosome fuses with the lysosome to form autolysosomes, which degrades the engulfed contents. (b) A model of mitophagy in yeast. Hog1 and Pbs2 regulate mitophagy by promoting the phosphorylation of Ser114 and Ser119 on Atg32. When Ser114 is phosphorylated, Atg32 binds to Atg11. Atg11 is a core component that facilitates the recruitment of specific cargoes to PAS, where the autophagosomes are generated. Atg32 also binds to Atg8, a factor that is essential for autophagosome formation. Autophagosomes envelop the mitochondria and fuse with lysosomes to degrade the mitochondria. (c) A model of mitophagy in mammalian cells. When mitochondria are depolarized, PINK1 accumulates on the mitochondrial outer membrane and recruits Parkin from the cytosol to the depolarized mitochondria in a manner dependent on the kinase activity of PINK1. The PINK1/Parkin complex triggers the autophagosome formation and the degradation of damaged mitochondria. Recent findings suggest that the proteasome is also involved in the destruction of mitochondria (see text for details).

 Autophagy is a bulk degradation system of cytoplasmic contents (Figure 2(a)) [25]. Upon induction of autophagy, a double-membrane compartment, termed the isolation membrane, engulfs a portion of cytoplasm, including macromolecules and organelles, and forms autophagosomes. The late autophagosome fuses with the lysosome to form autolysosomes, which degrades the engulfed contents. Autophagy is a highly conserved system among eukaryotes and important for many biological processes, such as cell survival during starvation, development, intracellular clearance, and immune responses. In the 1990s, genetic studies in yeast identified a series of autophagy-related (Atg) genes [26, 27]. The Atg proteins are involved in autophagosome formation and are divided into several functional groups [28]. Among them, Atg1 and its mammalian homologs Unc51-like kinase 1 and 2 (ULK1/2) function as the most upstream regulators of autophagosome formation in yeast and mammal, respectively. Under nutrient-rich conditions, the Atg1/ULK complex is suppressed by mammalian target of rapamycin complex 1 (mTORC1). Upon nutrient starvation, the Atg1/ULK complex is activated and recruited to autophagosome formation sites, such as pre-autophagosomal structure (PAS) in yeast and a specialized domain of the ER in mammals. At these sites, another functional unit, the class III phosphatidylinositol 3-kinase complex, produces phosphatidylinositol 3-phosphate (PI3P), and the PI3P-enriched membrane subdomain provides a platform for recruiting other molecules and thus for biogenesis of autophagosomes. The Atg proteins also function as critical components of the two ubiquitin-like conjugation systems; one is the Atg12 conjugation system that produces the Atg12-Atg5-Atg16 complex and the other is the Atg8/LC3-phosphatidylethanolamine (PE) conjugation system that plays important roles in the elongation and closure of the isolation membrane.

Although autophagy has been considered an essentially nonselective process, some proteins and organelles can be specifically recognized by autophagosomes. The autophagy-mediated selective degradation of organelles is thought to be a mechanism for eliminating damaged organelles to maintain cellular homeostasis. Because mitochondria are a major source of ROS, the harmful but inevitable byproduct of oxidative phosphorylation, it is especially important for the cell to monitor and regulate the quality and quantity of mitochondria. Mitophagy, the autophagy-mediated degradation of mitochondria, is one system that exists for this purpose [24]. Recent studies have identified the detailed molecular mechanisms responsible for the selectiveness of mitophagy. Moreover, it has been suggested that several kinases are involved in the precise regulation of mitophagy. In the following subsections, we focus on several kinases that regulate mitophagy in yeast and mammals.

### 3.1. Atg32 Is a Critical Regulator of Mitophagy in Yeast

 In yeast, two selective autophagy pathways have been well described: the cytoplasm-to-vacuole targeting (Cvt) pathway, which delivers vacuolar proenzymes, such as proaminopeptidase I and *α*-mannosidase, to the vacuole, and the pexophagy pathway, which induces the selective degradation of peroxisomes [29]. Recently, two groups have reported that mitochondria are also selectively degraded by autophagy under some growth conditions in yeast [30, 31]. They performed a genome-wide visual screen for mutants that are defective in mitophagy and identified Atg32 as a critical regulator of mitophagy in yeast (Figure 2(b)). Atg32 is a single membrane-spanning protein that is anchored on the mitochondrial surface. Atg32 binds to Atg11, which serves as an adaptor for the Cvt pathway and pexophagy by linking cargos to the autophagy machinery and to Atg8, which is essential for autophagosome formation. Atg32 binds to Atg8 through the WXXI/L/V motif in its N-terminus, a conserved motif that serves as a binding site for Atg8 family proteins. In the *atg32 Δ* strain, mitophagy is completely blocked, but other autophagy pathways, including nonselective macroautophagy and other types of selective autophagy, are not affected, indicating that Atg32 is mitophagy specific.

 The expression level of Atg32 is dramatically increased under mitophagy-inducing conditions in an ROS-dependent manner [30], indicating that Atg32-mediated mitophagy in yeast is regulated at least in part by Atg32 induction. In addition, recent studies have suggested that Ser114 and Ser119 on Atg32 are phosphorylated when mitophagy is induced [32]. Specifically, the phosphorylation of Atg32 at Ser114 is required for the Atg32-Atg11 interaction. Because Atg11 is known to recruit specific cargoes to the pre-autophagosomal structure (PAS), where the autophagosome is generated, the phosphorylation of Atg32 is required for the efficient delivery of mitochondria to the PAS. The phosphorylation of Atg32 at these sites is mediated by downstream components of the yeast mitogen-activated protein (MAP) kinase cascade, Hog1 and Pbs2 [32]. Although Hog1 and Pbs2 are activated in response to hyperosmotic stress and are involved in the osmoregulatory signal transduction cascade (the HOG signaling pathway), they are also activated under mitophagy-inducing conditions [33]. These findings suggest that mitophagy in yeast is strictly regulated through Atg32 phosphorylation, which is exerted by the interplay of cytosolic stress-responsive MAP kinases and the mitochondrial Atg32.

 Although the physiological role of mitophagy in yeast remains unclear, it has recently been reported that undegraded mitochondria in the *atg32 Δ* strain produce excess ROS, which cause additional mitochondrial damage and lead to even further accelerated ROS production. This vicious cycle ultimately results in the mitochondrial DNA deletion [34]. These findings suggest that mitophagy prevents excess ROS production by eliminating damaged mitochondria.

### 3.2. PINK1-Parkin-Dependent Mitophagy in Mammals

 Parkinson's disease (PD) is one of the most common neurodegenerative disorders. The causes of dopaminergic neuronal cell death in PD remains elusive, but many studies have suggested that mitochondrial dysfunction is likely to be an important factor. Specifically, the identification of the Ser/Thr kinase PINK1 (PTEN-induced putative kinase 1) as a gene responsible for early-onset autosomal recessive PD reinforced the link between mitochondria and PD because PINK1 is localized to the mitochondria [35]. Although it has been reported that PINK1 can phosphorylate some mitochondrial proteins either directly or indirectly, including the chaperone TRAP1 [36] and the protease HtrA2 [37], the function of PINK1 was not fully clear. A major breakthrough was made in a genetic study using *Drosophila melanogaster* [38]. This study revealed that PINK1 and the cytoplasmic E3 ligase Parkin, another gene responsible for early-onset autosomal recessive PD, function in the same pathway and that PINK1 functions upstream of Parkin. This study led to the discovery that PINK1 and Parkin cooperatively induce mitophagy to eliminate damaged mitochondria [39]. PINK1/Parkin-dependent mitophagy is only observed when cells are treated with the chemical reagent CCCP (carbonyl cyanide *m*-chlorophenyl hydrazone), which causes mitochondrial depolarization. Because CCCP disrupts the proton gradient across the mitochondrial inner membrane and thus dissipates the mitochondrial membrane potential (ΔΨm) that is required for oxidative phosphorylation, loss of mitochondrial membrane potential (ΔΨm) by CCCP treatment is regarded as a hallmark of damaged mitochondria. It has been reported that PINK1 is rapidly and constitutively degraded in healthy mitochondria but stabilized on the surface of mitochondria in response to ΔΨm (Figure 2(c)) [40–42]. Stabilized PINK1 acts as a marker of damaged mitochondria and recruits Parkin to the mitochondria [43]. Although the detailed mechanism by which the PINK1/Parkin complex recruits the autophagosome remains to be investigated, Parkin ubiquitinates substrates on mitochondria, leading to their proteasomal degradation and ultimately the destruction of the entire damaged mitochondria via autophagy [44, 45]. Importantly, many pathogenic mutations of PINK1 and Parkin fail to induce mitophagy, suggesting a model in which the impairment of PINK1/Parkin-mediated elimination of damaged mitochondria by mitophagy may contribute to PD pathogenesis [40, 41].

**Figure 3:**
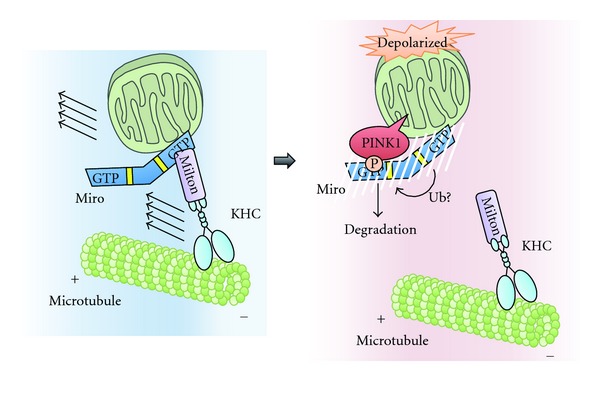
The mechanism of PINK1/Parkin-dependent arrest of mitochondrial motility. Long-range mitochondrial transport relies on the action of the molecular motor complex, which includes KHC, Milton, and Miro. When mitochondria are depolarized, stabilized PINK1 phosphorylates Ser156 of Miro. Subsequent interaction of Parkin with Miro and likely ubiquitination by Parkin causes proteasomal degradation of Miro to arrest mitochondrial motility.

 Interestingly, the kinase activity of PINK1 is required for the recruitment of Parkin to mitochondria and subsequent mitophagy [40, 41]. Although the substrate of PINK1 in the context of mitophagy has not been identified, a recent study has revealed that PINK1 regulates mitochondrial motility by phosphorylating Miro, a component of the motor/adaptor complex that mediates the axonal transport of mitochondria [46]. As mentioned in Section 2, mitochondria form a dynamic network, and the appropriate distribution of the mitochondrial network is important for many cellular functions, especially in highly polarized cells, such as neurons [47]. For example, in neurons, the appropriate positioning of mitochondria at the synapse is thought to be required to maintain local ATP levels for the generation of new synaptic branches [48]. Long-range mitochondrial transport from the soma to distal axonal and dendritic regions depends on the polarity and organization of neuronal microtubules as well as on the action of the molecular motor complex, which includes kinesin-1 heavy chain (KHC) (a motor protein), Milton (a kinesin-associated protein), and Miro (a mitochondrial Rho GTPase) (Figure 3) [48]. In this complex, Miro links directly to mitochondria, whereas Milton links indirectly to microtubule through KHC. PINK1 interacts with Miro in a CCCP-dependent manner and phosphorylates Ser156 on Miro, which promotes its degradation by the proteasome [46]. Interestingly, the CCCP-dependent degradation of Miro is promoted by the overexpression of Parkin, indicating that PINK1 and Parkin cooperatively regulate Miro expression. Whereas the PINK1/Parkin-mediated degradation of Miro completely blocks mitochondrial motility, the Miro S156A mutant is resistant to degradation and inhibits PINK1/Parkin-mediated mitochondrial arrest. Although the physiological relevance of CCCP-dependent mitochondrial arrest is not fully understood, at least two possibilities have been proposed: one, this arrest may prevent the mitochondrial fusion, and two, the arrest may sequester the damaged mitochondria and facilitate their efficient delivery to autophagosomes [46].

## 4. Mitochondria Are Platforms for Apoptosis Execution

 When cells undergo sufficient damage, apoptosis is induced. Apoptosis is a programmed process for eliminating damaged cells, and it is considered an important strategy for maintaining organismal homeostasis.

 All pathways to apoptosis converge on the activation of caspases, a family of cysteinyl aspartate proteases. There are two caspase families, the initiator caspases (caspase 8 and 9) and executor caspases (caspase 3, 6, and 7). Apoptosis is divided into two distinct pathways, depending on which initiator caspases are involved [49]. One pathway, known as the extrinsic pathway, is induced by cell surface death domain-containing receptors, such as Fas and tumor necrosis factor (TNF) receptors. In this pathway, caspase 8 is recruited to the cytoplasmic region of each receptor through the adaptor protein Fas-associated death domain (FADD) and subsequently activated. The second pathway, known as the intrinsic pathway, is activated by various cytotoxic insults, such as viral infection, DNA damage, and glucose deprivation. This pathway is also called the Bcl-2-regulated or mitochondrial pathway because the mitochondria play an important role in the activation of caspase 9, the initiator caspase of this pathway, and this step is strictly controlled by the Bcl-2 family of mitochondrial proteins [50].

**Figure 4:**
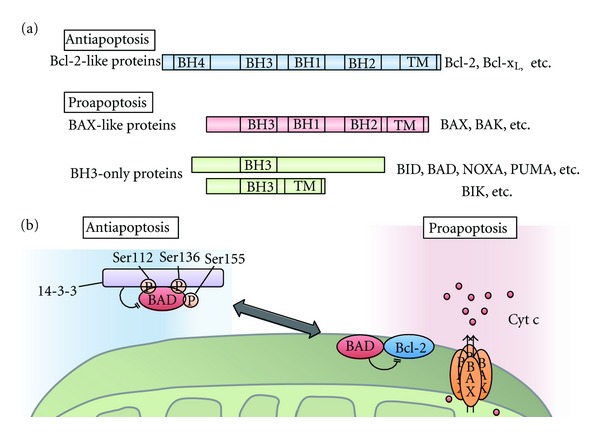
Bcl-2 family in apoptosis. (a) The domain structure of three classes of Bcl-2 family proteins. Antiapoptotic Bcl-2-like proteins have four Bcl-2 homology (BH) domains and a C-terminal transmembrane domain. Proapoptotic Bcl-2 family proteins are classified into two groups. Bax-like proteins have three BH domains (BH1-BH3) and a C-terminal transmembrane domain. Proapoptotic proteins, such as BID, BAD, NOXA, and PUMA, only have a BH3 domain and thus are called BH3-only proteins; some BH-3-only proteins, such as BIK, have a C-terminal transmembrane domain. (b) Survival signals suppress apoptosis by promoting the phosphorylation of BAD at Ser112, Ser136, and Ser155. When these sites are phosphorylated, BAD binds to 14-3-3 proteins, which suppresses the proapoptotic activity of BAD by inhibiting its association with antiapoptotic proteins such as Bcl-2. During apoptosis, dephosphorylated BAD dimerizes with Bcl-x_L_ or Bcl-2 and promotes the release of inner mitochondrial proteins such as cytochrome c (Cyt c) through pores formed by BAX.

 Bcl-2 family members have been grouped into three classes; Bcl-2-like proteins (e.g., Bcl-2 and Bcl-x_L_), which inhibit apoptosis, BAX-like proteins (e.g., BAX and BAK), which promote apoptosis, and the so-called BH3-only proteins (e.g., BAD, BID, PUMA, and NOXA), which are also proapoptotic but share only the BH3 domain with other Bcl-2 family members (Figure 4(a)).

 The proapoptotic effects of BAX and BAK are executed through their activities on mitochondria [50]. These proteins are thought to form pores in mitochondrial membranes and induce the release of soluble proapoptotic mitochondrial proteins, such as cytochrome c and Smac/DIABLO, into the cytosol. This process is called mitochondrial outer membrane permeabilization (MOMP). Once cytochrome c is released, it binds to APAF1 and leads to the assembly of a heptameric protein ring, the so-called “apoptosome,” that can bind to procaspase 9 and trigger its activation by promoting its self-cleavage [2]. Under normal conditions, antiapoptotic Bcl-2-like proteins, such as Bcl-2 and Bcl-x_L_, dimerize with proapoptotic BAX-like proteins, such as BAX and BAK, and suppress their proapoptotic activities [50]. However, once cells are exposed to apoptotic stimuli, BH3-only proteins are activated and sequester the antiapoptotic Bcl-2-like proteins from BAX and BAK, freeing BAX and BAK to carry out their proapoptotic functions.

 Because proapoptotic BH3-only proteins function as the initial sensors of diverse apoptotic stimuli, their activity must be precisely regulated. Some BH3-only proteins are known to be induced by specific transcription factors [50]. For example, NOXA and PUMA are induced by the tumor suppressor p53 in response to DNA damage. Other BH3-only proteins are activated posttranslationally [50]. For example, the caspase-8-mediated cleavage of BID downstream of death receptor signaling promotes the translocation of BID to the mitochondria, which leads to BAX/BAK activation. In addition, several lines of evidence have suggested that Bcl-2 family proteins are regulated by phosphorylation. In the following subsections, we focus on the phosphorylation-dependent regulation of Bcl-2 family proteins and their connection to the mitochondrial apoptosis pathway.

### 4.1. Phosphorylation-Dependent Regulations of BAD

 The balance between prosurvival and prodeath Bcl-2 family proteins determines whether a cell lives or dies. It has been reported that survival signals inhibit the proapoptotic activity of Bcl-2 family members and tilt the balance toward survival. BAD was originally identified as a Bcl-2 binding protein and was the first cell death component to be identified as a regulatory target of survival signaling [51]. BAD selectively dimerizes with Bcl-x_L_ or Bcl-2 and inhibits their antiapoptotic activity (Figure 4(b)). BAD contains at least three inhibitory phosphorylation sites, Ser112, Ser136, and Ser155 [52, 53]. These sites are phosphorylated in response to several survival-promoting cytokines, including IL-3, PDGF, IGF-1, and BDNF. The phosphorylation of BAD at Ser112 and Ser136 creates a 14-3-3 protein-binding consensus motif, promoting the interaction of BAD with 14-3-3 proteins [52]. The 14-3-3 proteins sequester BAD in the cytosol and inhibit its binding to the antiapoptotic proteins Bcl-x_L_ and Bcl-2, thereby suppressing the proapoptotic activity of BAD. Several kinases that mediate the phosphorylation of these sites have been identified, including RSK [54], PKA [55], Pak1/5 [56, 57], and Pim-1 [58] for Ser112; Akt [59], p70S6K [60], and Pak1 [56] for Ser136. As mentioned in Section 2.2, PKA is targeted to mitochondria by the mitochondria-tethered AKAP protein. PKA also phosphorylates BAD in a mitochondrial AKAP-dependent manner [55]. The identification of another phosphorylation site, Ser155, on BAD led to the elucidation of a more detailed mechanism of BAD inactivation [53]. The recruitment of 14-3-3 proteins to BAD increases the access of survival-promoting kinases to BAD Ser155 (Figure 4(b)). The phosphorylation of Ser155 is considered to permanently inhibit the dimerization of BAD and the antiapoptotic proteins Bcl-x_L_ or Bcl-2. To examine the physiological roles of these inhibitory phosphorylation sites of BAD *in vivo*, Datta et al. generated knock-in mice harboring three BAD point mutations: S112A, S136A, and S155A [61]. These mice exhibit reduced numbers of pro-lymphocytes of the T-cell and B-cell lineage because their survival-promoting cytokine IL-7 is unable to suppress the proapoptotic activity of BAD. Moreover, primary cultured cells derived from these mutant mice are hypersensitive to apoptosis-inducing stimuli because of increased cytochrome c release from the mitochondria. These findings indicate that the survival factor-dependent phosphorylation of BAD raises the threshold at which mitochondria release cytochrome c in response to apoptotic stimuli [61].

 Although these phosphorylations inhibit BAD proapoptotic function, it has been suggested that BAD phosphorylation at Ser128 promotes apoptosis [62, 63]. Ser128 has been reported to be phosphorylated by the stress-responsive MAP kinase c-Jun N-terminal kinase (JNK) [62] and the cell cycle-regulated kinase Cdc2 [63]. When Ser128 is phosphorylated, the interaction of BAD with 14-3-3 proteins is disrupted, promoting the apoptotic effect of BAD [63]. It has also been reported that JNK exerts its proapoptotic function by phosphorylating Ser184 on 14-3-3 and promoting the dissociation of 14-3-3 from BAD [64]. In addition, the proapoptotic activity of BAD has been reported to be enhanced by dephosphorylation. The calcineurin-mediated dephosphorylation of BAD induces the translocation of BAD to the mitochondria, promoting its dimerization with Bcl-x_L_ and subsequent BAD-mediated apoptosis [65].

### 4.2. The Phosphorylation-Dependent Regulation of Bcl-2

In addition to the proapoptotic protein BAD described above, the antiapoptotic protein Bcl-2 is also regulated by phosphorylation. In particular, the phosphorylation of Bcl-2 induced by microtubule inhibitors has been examined. Haldar et al. first reported that Bcl-2 was phosphorylated in response to taxol, a well-studied microtubule-damaging agent [66]. Later, Thr69, Ser70, and Ser87 were found to be phosphorylated in cells treated with taxol [67]. When these residues were substituted with alanine, the antiapoptotic activity of Bcl-2 was augmented. Several reports support the hypothesis that the phosphorylation of Bcl-2 suppresses its antiapoptotic activity. However, other reports argue that the phosphorylation of Bcl-2 at Ser70 promotes its antiapoptotic activity [68]. Unfortunately, no sufficient explanation for this discrepancy has been provided to date.

 Because other microtubule inhibitors, such as vincristine, vinblastine, and nocodazole, also induce Bcl-2 phosphorylation, a relationship between the cell cycle and Bcl-2 phosphorylation was proposed. Furthermore, it has been reported that Bcl-2 Ser70 is phosphorylated by JNK1 in G2/M phase [67]. Although it is not clear why the antiapoptotic activity of Bcl-2 must be suppressed at G2/M, one possibility is that lowering the threshold for apoptosis might ensure the elimination of cells with aberrant chromosomal segregation.

## 5. PGAM5 Is a Mitochondrial Ser/Thr Protein Phosphatase That Regulates Multiple Cell Death Pathways

 In addition to the mitochondrial kinases, there are also mitochondrial phosphatases; we identified phosphoglycerate mutase family member 5 (PGAM5) as a novel Ser/Thr protein phosphatase in the mitochondria [69]. We identified PGAM5 as a binding protein of apoptosis-signal regulating kinase 1 (ASK1), a stress-responsive MAPKKK that activates the JNK and p38 MAPK pathways. PGAM5 belongs to the PGAM family, an evolutionarily conserved family of enzymes that convert 3-phosphoglycerate to 2-phosphoglycerate during glycolysis [70]. Although a common catalytic domain, the PGAM domain, is also conserved in PGAM5, we found that PGAM5 lacks mutase activity and instead acts as a Ser/Thr protein phosphatase. When the conserved histidine residue that is known to function as a phospho-acceptor in the mutase reaction is disrupted by mutagenesis, PGAM5 completely loses its phosphatase activity. Moreover, the phosphatase activity of PGAM5 is required for the activation of stress-responsive MAP kinase pathways. Together with the previous report that PGAM5 is localized in mitochondria through its N-terminal transmembrane domain [71], these findings indicate that PGAM5 is a novel mitochondria-resident Ser/Thr protein phosphatase that might be involved in the mitochondrial and/or cellular stress responses [69].

PGAM5 was also identified as a novel substrate of the Keap1-dependent ubiquitin ligase complex [71, 72]. Keap1 is a BTB-Kelch substrate adapter for the Cul3-dependent E3 ubiquitin ligase complex. The best-studied substrate of Keap1 is Nrf2, a transcription factor that regulates the expression of various cellular redox state-regulating proteins, including antioxidant enzymes. Under normal conditions, Nrf2 is constitutively degraded in a Keap1-dependent manner. However, oxidative stress modifies some cysteine residues on Keap1 and disrupts the formation of a functional E3-ligase complex, leading to the stabilization of Nrf2. The N-terminus of PGAM5 contains a Keap1-binding consensus motif, E(S/T)GE, similar to that found in Nrf2. Although it is not known why PGAM5 is degraded in a Keap1-dependent manner, the notion that mitochondria are a major source of ROS raises the possibility that the Keap1-PGAM5 complex might be involved in mitochondrial redox homeostasis.

 The PGAM5 protein is highly conserved in many species, including *Drosophila  melanogaster *and *Caenorhabditis elegans*. Recently, the *Drosophila* ortholog of mammalian PGAM5 (dPGAM5) has been shown to exacerbate mitochondrial degeneration and dopaminergic neuronal cell death in a model of Parkinson's disease induced by mutation of the *Drosophila PINK1 *gene [73]. Because PINK1 has been shown to play a critical role in the induction of mitophagy, as mentioned in Section 3, the genetic interaction of PINK1 and PGAM5 suggests that PGAM5 might be involved in mitochondrial quality control.

PGAM5 has several unique and interesting features, as mentioned above. Although its physiological functions have been largely unknown, recent findings have suggested that PGAM5 functions as a regulator of multiple cell death pathways. In the following subsections, we will summarize the recent reports on PGAM5 as a cell death regulator.

### 5.1. PGAM5 in Necroptosis

 Necrosis is one type of cell death that is morphologically distinct from apoptosis [6]. During apoptosis, cells break into small membrane-wrapped vesicles known as apoptotic bodies. In contrast, necrosis is characterized by a discontinuous cytoplasmic membrane and organelle swelling. Until recently, apoptosis was considered to be the sole form of programmed cell death, whereas necrosis was regarded as an unregulated and uncontrollable process. However, the recent identification of components that transduce necrosis-specific signals suggests that necrosis is also a regulated process. Following this finding, necrosis came to be defined as “necroptosis.” Several more additional recent studies have revealed that necroptosis participates or is involved in the pathogenesis of various diseases, including ischaemic injury, neurodegeneration, and viral infection.

**Figure 5:**
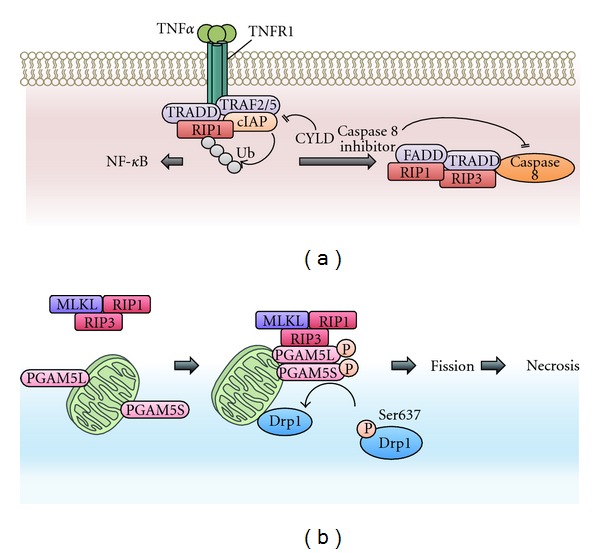
Necrosis signaling pathways. (a) Necrosis is induced downstream of death domain-containing receptors, including TNF receptors 1 (TNFR1) and TNFR2. When TNF*α* binds to TNFR1, RIP1 is recruited to the activated TNFR1. In this receptor-associated complex, E3 ligase cellular inhibitor of apoptosis proteins (cIAPs) mediate the Lys63-linked polyubiquitin of RIP1, activating the NF-*κ*B pathway. Conversely, when the Lys63-linked polyubiquitin is removed by deubiquitinating enzymes, including CYLD, RIP1 functions as a cell death inducer rather than as a survival-promoting factor. When caspase 8 activity is blocked by inhibitors, such as a pan-caspase inhibitor or Z-VAD-fmk, or by the genetic ablation of caspase 8 itself, RIP1 interacts with RIP3 and forms a necrosis-inducing complex. RIP1 and RIP3 become phosphorylated and activated in this complex. Although the kinase activity of RIP1 is not required to activate the NF-*κ*B pathway, its kinase activity is essential for necrosis induction. (b) The mitochondrial Ser/Thr protein phosphatase PGAM5 is a substrate of RIP3. The RIP3-mediated phosphorylation of PGAM5 enhances its protein phosphatase activity. Once activated by phosphorylation, PGAM5 dephosphorylates and activates the mitochondrial fission regulator Drp1, promoting mitochondrial fission and subsequent necrosis. PGAM5 has two splice variants in humans, PGAM5L and PGAM5S, each of which appears to have different functions in necrosis (see text for details).

 Necroptosis is induced by the ligation of death domain-containing receptors, including TNF receptor 1 (TNFR1) and TNFR2, under specific conditions in which caspases are inhibited [6]. The main components of the necroptosis signaling pathway are two Ser/Thr kinases, receptor-interacting protein 1 (RIP1), and RIP3 [74–76]. Upon necrosis-inducing stimuli, RIP1 and RIP3 interact with each other to form a complex (Figure 5(a)). Because the kinase activity of RIP1 and RIP3 is essential for the execution of necroptosis, the identification of their substrates has been a major focus of recent research. The pharmacological inhibition of RIP1 abolishes the recruitment of RIP3 and thereby inhibits RIP3 activation, suggesting that RIP1 is upstream of RIP3 [75]. Although the substrates of RIP3 have been remained elusive, recent studies have uncovered some critical factors that act downstream of RIP3 in necroptosis. Interestingly, one of these proteins is PGAM5 [77] (Figure 5(b)). PGAM5 has two splice variants in humans, PGAM5L and PGAM5S. Upon exposure to necroptosis-inducing stimuli, both forms of PGAM5 are phosphorylated by RIP3 [77]. The RIP3-mediated phosphorylation of PGAM5 enhances its protein phosphatase activity. As mentioned in Section 2, mitochondrial fission has been implicated in cell death. Interestingly, PGAM5S appears to activate Drp1, most likely through dephosphorylation of the inhibitory Ser637 site of Drp1 and thereby promotes mitochondrial fission. Although it remains elusive how mitochondrial fission promotes necroptosis, the pronecrotic role of PGAM5 may be exerted in part by the regulation of mitochondrial fission [77].

### 5.2. PGAM5 in Apoptosis

Some recent reports have also revealed the involvement of PGAM5 in apoptosis. The comprehensive screening of Bcl-x_L_-binding proteins identified PGAM5 as one Bcl-x_L_ interactor [78]. Recently, it has been reported that PGAM5 bridges Keap1 and Bcl-x_L_ and facilitates the Keap1-E3 ligase complex-mediated degradation of Bcl-x_L_. Because the Keap1-dependent degradation of antiapoptotic Bcl-x_L_ protein enhances etoposide-induced apoptosis, PGAM5 appears to serve a proapoptotic function [79].

 However, our recent analysis of dPGAM5-deficient flies has revealed the antiapoptotic function of PGAM5 [80]. Null mutants of *dPGAM5* exhibited increased vulnerability to heat shock stress. Interestingly, dPGAM5 deficiency in the mushroom body, a brain structure that plays a central role in higher brain functions such as olfactory learning and memory, is sufficient for vulnerability to heat shock stress, indicating that dPGAM5 in the mushroom body plays a critical role in whole-body response to heat shock stress. Importantly, phosphatase-inactive dPGAM5 could not rescue this vulnerability, indicating that the role of dPGAM5 in heat shock response is dependent on its phosphatase activity. Moreover, after heat shock treatment, apoptotic cells are detected in the mushroom body of dPGAM5-deficient flies but not in wild-type flies, indicating that dPGAM5 protects against heat shock stress by preventing apoptosis in the mushroom body. Although the detailed molecular mechanisms through which dPGAM5 prevents apoptosis against heat shock stress are not known, we currently consider that dPGAM5 might sense heat shock stress-induced mitochondrial defects, such as the accumulation of heat-damaged proteins within mitochondria, and transduce signals from mitochondria to other cellular compartments [80].

## 6. Conclusions

 Here, we have discussed the phosphorylation-dependent regulation of mitochondrial proteins in the mitochondrial and cellular stress responses. Mitochondrial protein phosphorylation contributes to the regulation of mitochondrial dynamics and mitophagy, which are important to maintain mitochondrial quality and ultimately ensure cellular homeostasis. Furthermore, mitochondrial protein phosphorylation is also involved in cell stress-induced programmed cell death, such as apoptosis and necroptosis. Further studies of mitochondrial protein phosphorylation will lead to a better understanding of the mitochondrial and cellular stress responses and related pathophysiological phenomena.
